# Basal gonadotropin levels combine with pelvic ultrasound and pituitary volume: a machine learning diagnostic model of idiopathic central precocious puberty

**DOI:** 10.1186/s12887-023-04432-0

**Published:** 2023-11-29

**Authors:** Tao Chen, Danbin Zhang

**Affiliations:** Department of Radiology, The First Affiliated Hospital, College of Medicine, Zhejiang University, 79 Qingchun Road, Hangzhou, 310003 Zhejiang China

**Keywords:** Central precocious puberty, Machine learning, Magnetic resonance imaging, Pelvic Ultrasound, Uterine volume

## Abstract

**Objective:**

The current diagnosis of central precocious puberty (CPP) relies on the gonadotropin-releasing hormone analogue (GnRHa) stimulation test, which requires multiple invasive blood sampling procedures. The aim of this study was to construct machine learning models incorporating basal pubertal hormone levels, pituitary magnetic resonance imaging (MRI), and pelvic ultrasound parameters to predict the response of precocious girls to GnRHa stimulation test.

**Methods:**

This retrospective study included 455 girls diagnosed with precocious puberty who underwent transabdominal pelvic ultrasound, brain MRI examinations and GnRHa stimulation testing were retrospectively reviewed. They were randomly assigned to the training or internal validation set in an 8:2 ratio. Four machine learning classifiers were developed to identify girls with CPP, including logistic regression, random forest, light gradient boosting (LightGBM), and eXtreme gradient boosting (XGBoost). The accuracy, sensitivity, specificity, positive predictive value, negative predictive value, area under receiver operating characteristic (AUC) and F1 score of the models were measured.

**Results:**

The participates were divided into an idiopathic CPP group (n = 263) and a non-CPP group (n = 192). All machine learning classifiers used achieved good performance in distinguishing CPP group and non-CPP group, with the area under the curve (AUC) ranging from 0.72 to 0.81 in validation set. XGBoost had the highest diagnostic efficacy, with sensitivity of 0.81, specificity of 0.72, and F1 score of 0.80. Basal pubertal hormone levels (including luteinizing hormone, follicle-stimulating hormone, and estradiol), averaged ovarian volume, and several uterine parameters were predictors in the model.

**Conclusion:**

The machine learning prediction model we developed has good efficacy for predicting response to GnRHa stimulation tests which could help in the diagnosis of CPP.

## Introduction

Precocious puberty in girls is defined as the onset of secondary sexual characteristics before the age of 8 and can be divided into three types: central precocious puberty (CPP), peripheral precocious puberty and incomplete precocious puberty [1, 2]. CPP results from the premature activation of the hypothalamic-pituitary-gonadal (HPG) axis. About 90% of cases in girls are idiopathic without definite organic disease [3]. Idiopathic CPP (ICPP) may mimic other forms of precocious puberty and can lead to short stature in adults due to early epiphyseal fusion, and adverse psychosocial outcomes. Thus, it is very important to diagnosis ICPP in subjects with early symptoms of puberty [3, 4].

To date, the gonadotropin-releasing hormone analogue (GnRHa) stimulation test is considered the gold standard to distinguish between the intermediate forms of precocious puberty that are not suitable for treatment with GnRHa and CPP [5, 6]. However, the GnRHa stimulation tests require multiple invasive blood sampling procedures which is inconvenient in paediatric patients [7, 8]. Pelvic ultrasound, as rapid and non-invasive tests, is currently routine examinations utilized in female patients with precocious puberty. Pelvic ultrasound is considered an additional tool in the diagnosis of CPP in a situation when the results of the GnRH stimulation test are opaque [9]. Magnetic resonance imaging (MRI) of the brain can be used to determine the presence of brain lesions causing premature pubertal development. [10, 11]. The possibility of replacing the GnRHa stimulation test with basal pubertal hormones, such as luteinizing hormone (LH) and routine imaging tests has been continuously reviewed [12–14]. Nevertheless, the consensus on its use in the case of suspected ICPP has not yet been established [3].

Considering the extensive application of machine learning classifiers in the medical field, we aimed to construct models based on basal pubertal hormone levels, pituitary dimensions measured by MRI, and pelvic ultrasound parameters using various machine learning classifiers to diagnose girls with ICPP.

## Materials and methods

### Participants

**Fig. 1:**
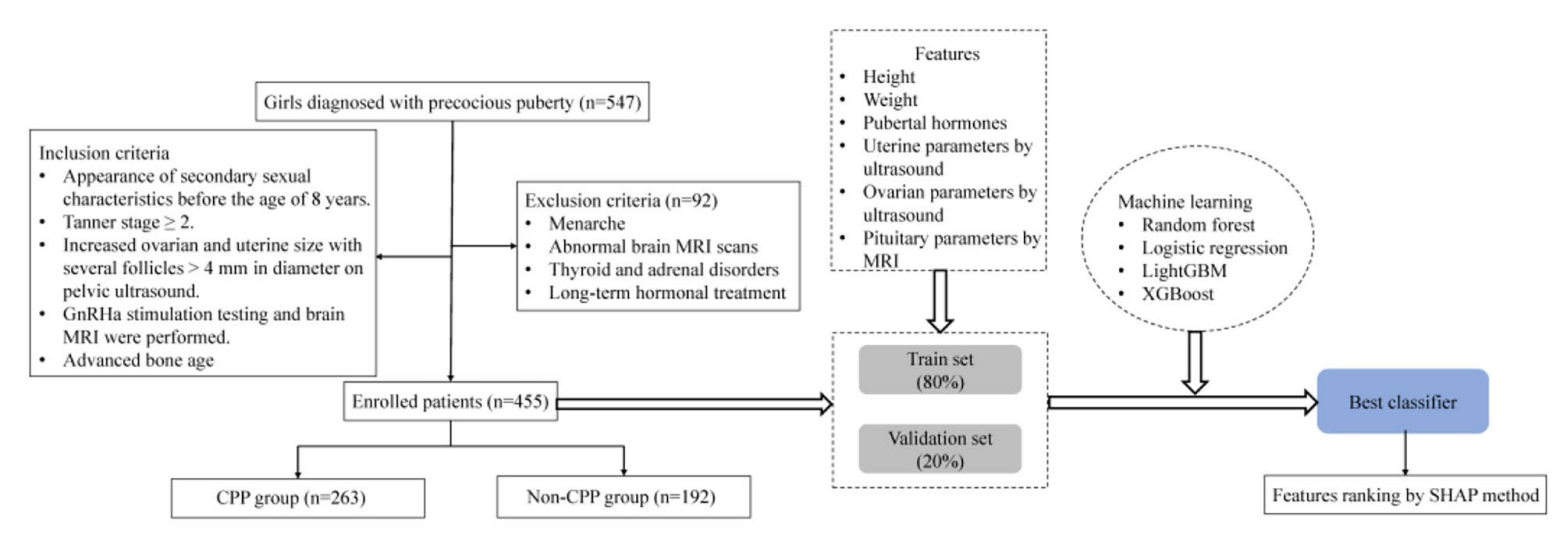
Flowchart of the study cohort Abbreviations: GnRHa: Gonadotropin-releasing hormone analogue; CPP: Central precocious puberty; MRI: Magnetic resonance imaging; LightGBM: light gradient boosting, XGBoost: eXtreme gradient boosting; SHAP: SHapley Additive exPlanations

We retrospectively reviewed the medical charts of all pediatric female patients (age 4 − 10 years) who diagnosed with precocious puberty in the First Affiliated Hospital, Zhejiang University School of Medicine between January 2018 and December 2022. The inclusion criteria are as follows: (a) appearance of secondary sexual characteristics before the age of 8 years and lasting more than 3 months (such as increased growth velocity, breast or pubic hair development), (b) Tanner stage ≥ 2, (c) increased ovarian and uterine size with several follicles > 4 mm in diameter on pelvic ultrasound, (d) GnRHa stimulation testing and brain MRI were performed, (e) advanced bone age. The exclusion criteria were: (a) menarche, (b) abnormal pituitary or brain MRI scans, (c) thyroid and adrenal disorders, and (d) long-term hormonal treatment. Finally, 455 participants were enrolled in this study (Fig. 1).

The study was approved by the Ethics Committee of the First Affiliated Hospital, Zhejiang University School of Medicine.

### GnRHa stimulation test and hormonal measurement

The basal LH, follicular-stimulating hormone (FSH) and estradiol levels were measured by immuno-chemiluminescence assay on all participants in a fasting state between 8:00 am and 8:30 am. The detection limits of basal LH and FSH were 0.01 and 0.05 IU/L, respectively. Triptorelin acetate was injected, with a dosage of 2.5 ug/kg, a maximum dosage of 100 ug. About 2 mL of blood was collected at each time point after injection (30, 60, and 90 min). The concentrations of LH and FSH were determined in each sample. Patients with a peak LH value > 5.0 IU/L and peak LH/FSH ratio > 0.6 were defined as CPP [2], others were included in the non-CPP group.

### Pelvic ultrasound

Transabdominal pelvic ultrasound scans were performed in all participants before the GnRHa stimulation test by experienced ultrasound physicians. They were obtained with a Philips P700 ultrasound unit, (Philips Medical Systems Inc., Bothell, WA) equipped with a 5 MHz convex-array broadband transducer or a 7.5 MHz linear-array small parts transducer, depending on the patient. To create an acoustic window, all participants were required to drink water to ensure a full bladder. The reported parameters for the uterus included the length, transverse diameter, anteroposterior diameter and the presence or absence of endometrial echogenicity. The cervical anteroposterior diameters were measured in the standard midsagittal view image of the uterus by two radiologists independently. The ratio between the fundal and cervical diameters (FCR) was calculated. In ovaries, the height, width, and length were evaluated. Both uterine volume and ovarian volume were calculated based on the ellipse formula (0.5233 × length × height × width). The average of the values for both ovaries was calculated for each patient.

### MRI acquisition and analysis

**Fig. 2:**
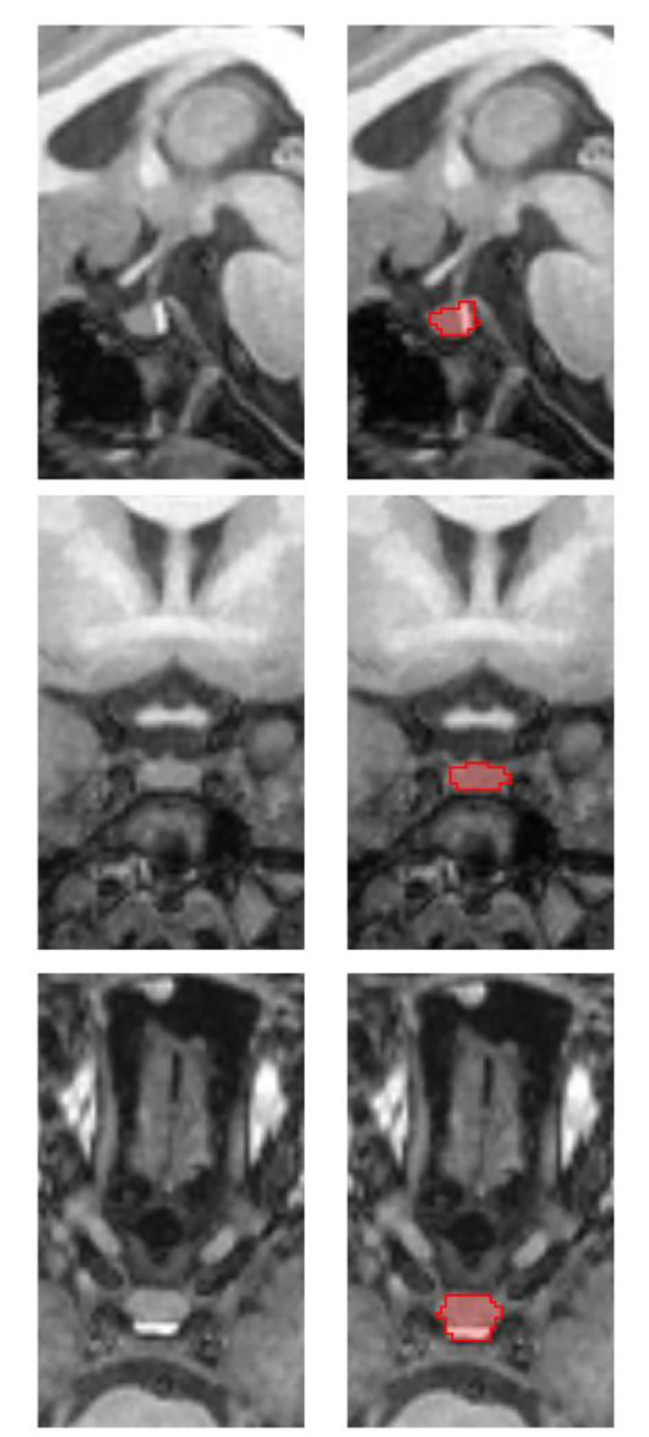
Sagittal (top), coronal (middle), and axial (bottom) view of the pituitary gland from a T1-weighted MR image. The pituitary is manually labelled in red on the right

Brain MRI was performed on a 3.0 T MRI scanner with an eight-channel phased head coil before the GnRHa stimulation test. The pituitary volume of each participant was determined manually (see Fig. 2) by a trained radiologists using the multi-image analysis software MANGO (Research Imaging Centre, UTHSCSA; http://ric.uthscsa.edu/mango). The definition and segmentation of the pituitary gland were based on previously published methods that included the posterior and anterior pituitary but excluded the pituitary stalk [15, 16]. It has been determined that the pituitary gland is best tracked in the coronal plane. Inferiorly, the sphenoid sinus defined the pituitary margin and the diaphragma sellae marked the border superiorly. The cavernous sinuses were used as bilateral borders [15]. Pituitary volume was estimated by adding up all the voxels in each tracking region in mm^3^. The region of interest (ROI) profile of the pituitary gland was determined by two experienced neuroradiologists independently. Both observers were blinded to the clinical data.

According to the Elster’s grade [17], we evaluated the pituitary morphology by observing the outline of the superior surface of the gland in the midsagittal plane. We simplified the original grade 5 (grade 1 = significant concavity, grade 2 = mild concavity, grade 3 = flat, grade 4 = mild concavity, grade 5 = significant concavity) to grade 3 (grade 1 = concave, grade 2 = flat, grade 3 = convex).

### Statistical analysis

Normally distributed data were shown as mean ± standard deviation. An independent sample t-test or Mann-Whitney *U* test was used to compare differences between the ICPP and non-CPP groups, as appropriate. Variables with statistical differences were included in further analysis. To select the classifier prediction model with the highest discrimination between ICPP and non-CPP group, we selected four machine learning classifiers including logistic regression, random forest, light gradient boosting (LightGBM), and eXtreme gradient boosting (XGBoost). We applied the 5-fold internal cross-validation to explore the optimal hyperparameters. Subsequently, the SHapley Additive exPlanations (SHAP) model interpretation method was used to individually calculate and analyse how each feature affected the output of the best classifier. All models were validated in the validation cohort. We plotted receiver operating characteristic curve (ROC) and compared the area under curve (AUC); then plotted calibration curve and clinical decision curve analysis (DCA) to quantify and compare the differences in clinical gain between the classifiers; furthermore, we calculated the classifier’s accuracy, sensitivity, specificity, positive predictive value (PPV), negative predictive value (NPV) and F1 score.

The interclass correlation coefficient (ICC) values of pituitary variables and the cervical anteroposterior diameter were calculated to evaluate the strength of interobserver agreement between the two radiologists (0.00-0.20 poor agreement, 0.21–0.40 fair agreement, 0.41–0.60 moderate agreement, 0.61–0.80 good agreement, and 0.81-1.00 excellent agreement). Correlation analyses were conducted to assess the relationship between all imaging parameters and pubertal hormone levels in the ICPP group. Differences were considered statistically significant when the two-tailed *p* value was < 0.05. All the statistical analyses were performed using SPSS 27.0 and Python software (version 3.11).

## Results

### Descriptive statistics

The ICPP group showed higher serum LH, FSH, LH/FSH ratio, estradiol levels, weight, height, and Tanner stage for breast development than the non-CPP group (all *P* < 0.05). No significant differences were observed in PRL, age, BMI, and Tanner stage for pubic hair development between the two groups.

The uterine length, transverse diameter of uterine, anteroposterior diameter of uterine, uterine volume, ratio of fundus to cervix, presence of endometrium, and average ovarian volume were found to be significantly higher in the ICPP group than in the non-CPP group (all *P* < 0.05). No significant differences were observed in the cervical anteroposterior diameter, ovarian height, length, and width, pituitary height, and pituitary volume between the two groups (see Table 1). The pituitary variables and the cervical anteroposterior diameter showed excellent agreement (ICC>0.9). No subjects were excluded because of poor image quality.

**Table 1:** Clinical characteristics of participants

Variables	ICPP group (n = 263)	non-CPP group (n = 192)	*P* value
Age, years	8.15 ± 1.17	8.21 ± 1.20	0.259
BA-CA, years	1.89 ± 1.62	1.40 ± 1.03	0.243
Weight, kg	29.64 ± 5.74	28.41 ± 5.59	0.023
Height, cm	130.24 ± 7.79	132.09 ± 7.28	0.010
BMI, kg/m^2^	16.86 ± 2.09	16.62 ± 1.95	0.204
Tanner stage (breast)	2.56 ± 0.71	2.19 ± 0.56	< 0.001
Tanner stage (pubic hair)	1.29 ± 0.44	1.15 ± 0.43	0.824
Baseline LH, IU/L	1.04 ± 1.54	0.34 ± 0.64	< 0.001
Baseline FSH, IU/L	3.86 ± 2.48	2.86 ± 1.76	< 0.001
Baseline LH/FSH ratio	0.39 ± 1.42	0.10 ± 0.12	0.001
Baseline Estradiol, pg/mL	28.61 ± 17.80	24.84 ± 13.70	0.011
PRL, ng/mL	11.10 ± 5.74	10.83 ± 6.02	0.618
Pituitary			
Height, mm	5.18 ± 1.11	4.93 ± 1.16	0.080
Volume, mm^3^	356.52 ± 113.41	343.92 ± 85.28	0.178
Shape grade	2.02 ± 0.60	1.96 ± 0.58	0.256
Uterine			
Length, mm	22.50 ± 5.51	19.85 ± 4.35	< 0.001
Anteroposterior diameter, mm	14.88 ± 5.55	13.20 ± 4.95	< 0.001
Transverse diameter, mm	11.06 ± 4.60	8.81 ± 3.39	< 0.001
Uterus volume, mL	2.45 ± 2.95	1.48 ± 1.64	< 0.001
Cervical			
Anteroposterior diameter, mm	11.09 ± 3.25	10.57 ± 3.04	0.085
FCR	1.34 ± 0.23	1.24 ± 0.23	< 0.001
Presence of endometrium, n (%)	180 (68.44)	101 (52.60)	< 0.001
Ovary			
Length, mm	24.87 ± 4.33	23.91 ± 3.38	0.215
Width, mm	12.01 ± 2.75	11.50 ± 2.31	0.286
Height, mm	14.09 ± 3.32	14.37 ± 4.21	0.653
Average volume, mL	2.31 ± 1.09	2.03 ± 0.94	0.004

ICPP: idiopathic central precocious puberty; LH: Luteinizing hormone; FSH: Follicular-stimulating hormone; PRL: prolactin; BA, bone age; CA, chronological age; BMI, body mass index; FCR, Ratio of fundus to cervix

### Development and validation of machine learning classifiers

**Fig. 3:**
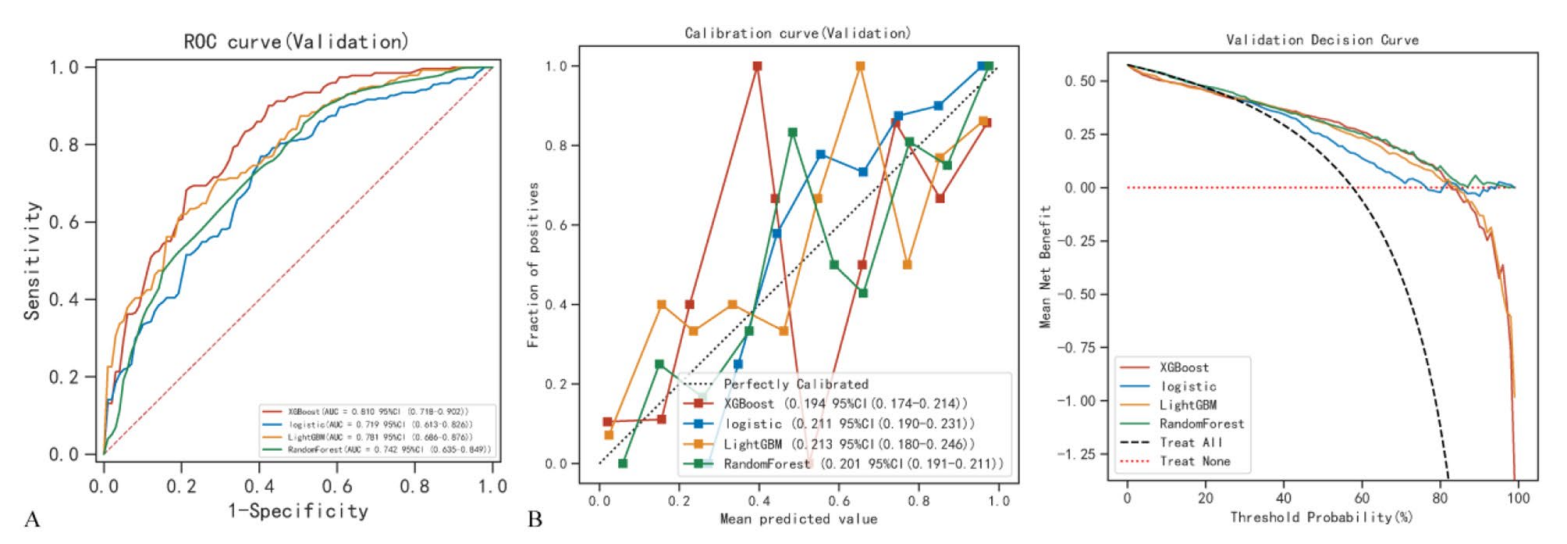
ROC curves (**A**), plotted calibration curves (**B**) and DCA curves (**C**) for four machine learning classifiers in the validation set Abbreviations: LightGBM, light gradient boosting; XGBoost, eXtreme Gradient Boosting

We used four machine learning algorithms, random forest, logistic regression, LightGBM, and XGBoost, to build the prediction models with the training dataset. The ROC, calibration curve and DCA of the prediction models based on different classifiers in the validation cohort were shown in Fig. 3; Table 2. All classifiers performed well in each dataset. The XGBoost shows the highest AUC value in the validation set.

**Table 2:** Diagnostic performance of different machine learning classifiers in validation cohort

Model	AUC	Accuracy	Sensitivity	Specificity	PPV	NPV	F1 score
XGBoost	0.81 (0.72–0.90)	0.68	0.81	0.72	0.80	0.59	0.80
LightGBM	0.78 (0.69–0.88)	0.69	0.72	0.76	0.77	0.60	0.73
Logistic	0.72 (0.61–0.83)	0.64	0.79	0.61	0.72	0.55	0.75
RandomForest	0.74 (0.70–0.86)	0.67	0.76	0.64	0.74	0.59	0.74

AUC, area under curve; LightGBM, light gradient boosting; NPV, negative predictive value; PPV, positive predictive value; XGBoost, eXtreme Gradient Boosting

### Visualization of feature importance for the best classifier

**Fig. 4:**
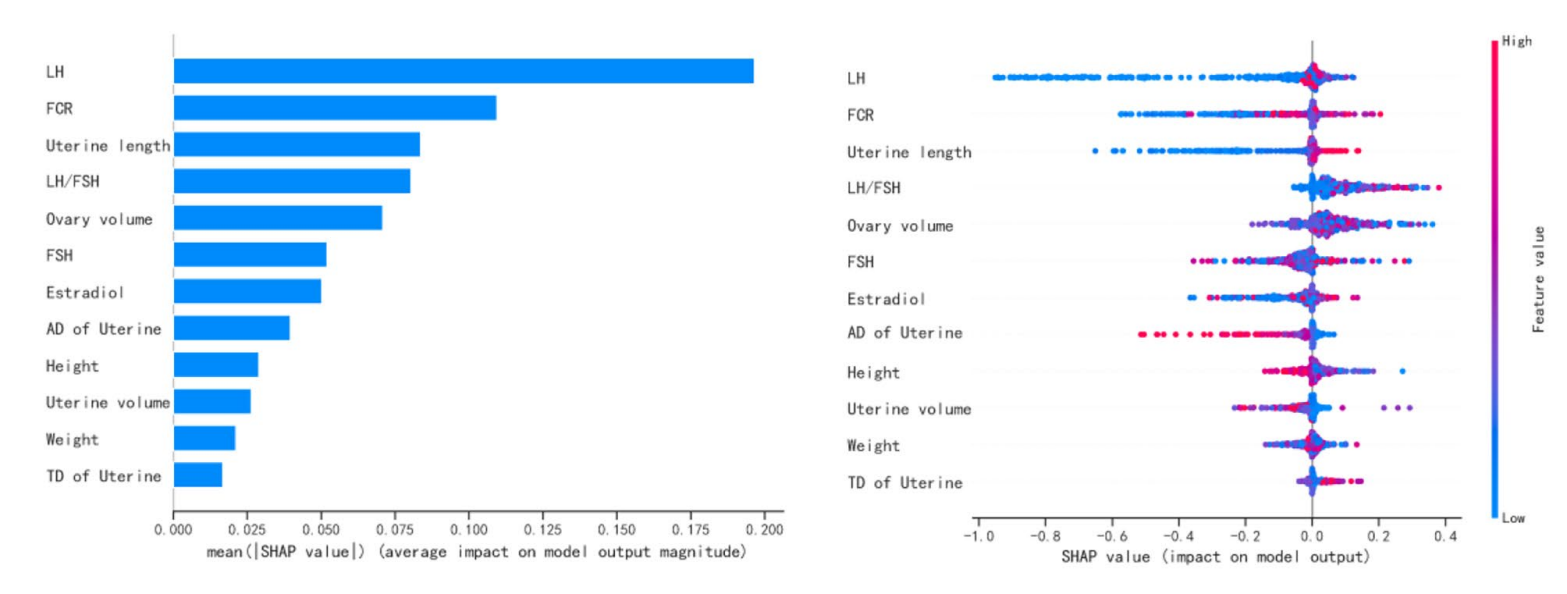
Summary plot of the importance of features in LightGBM classifier. Y-axis represents the importance of the features, in descending order. x-axis represents the contribution, where > 0 is a positive contribution and < 0 is a negative contribution. The color of the scatter indicates whether the feature is high (red) or low (blue) Abbreviations: LH: Luteinizing hormone; FSH: Follicular-stimulating hormone; TD, Transverse diameter; AD, Anteroposterior diameter; FCR, Ratio of fundus to cervix

To visually explain the features included in the XGBoost, we used SHAP to explain the role of these features in differentiating ICPP and non-ICPP in the model (Fig. 4). The SHAP values (x-axis) are a uniform quantification of the impact of the features included in the model, and the impact on the results is plotted with two coloured dots. The red dots represent high-risk values, and the blue ones represent low-risk values. The top 10 features were LH levels, ratio of fundus to cervix, uterine length, LH/FSH ratio, average ovarian volume, FSH levels, estradiol levels, anteroposterior diameter of uterine, height, and uterine volume.

### Correlations of pituitary volume, ultrasound parameters, and pubertal hormone levels

**Fig. 5:**
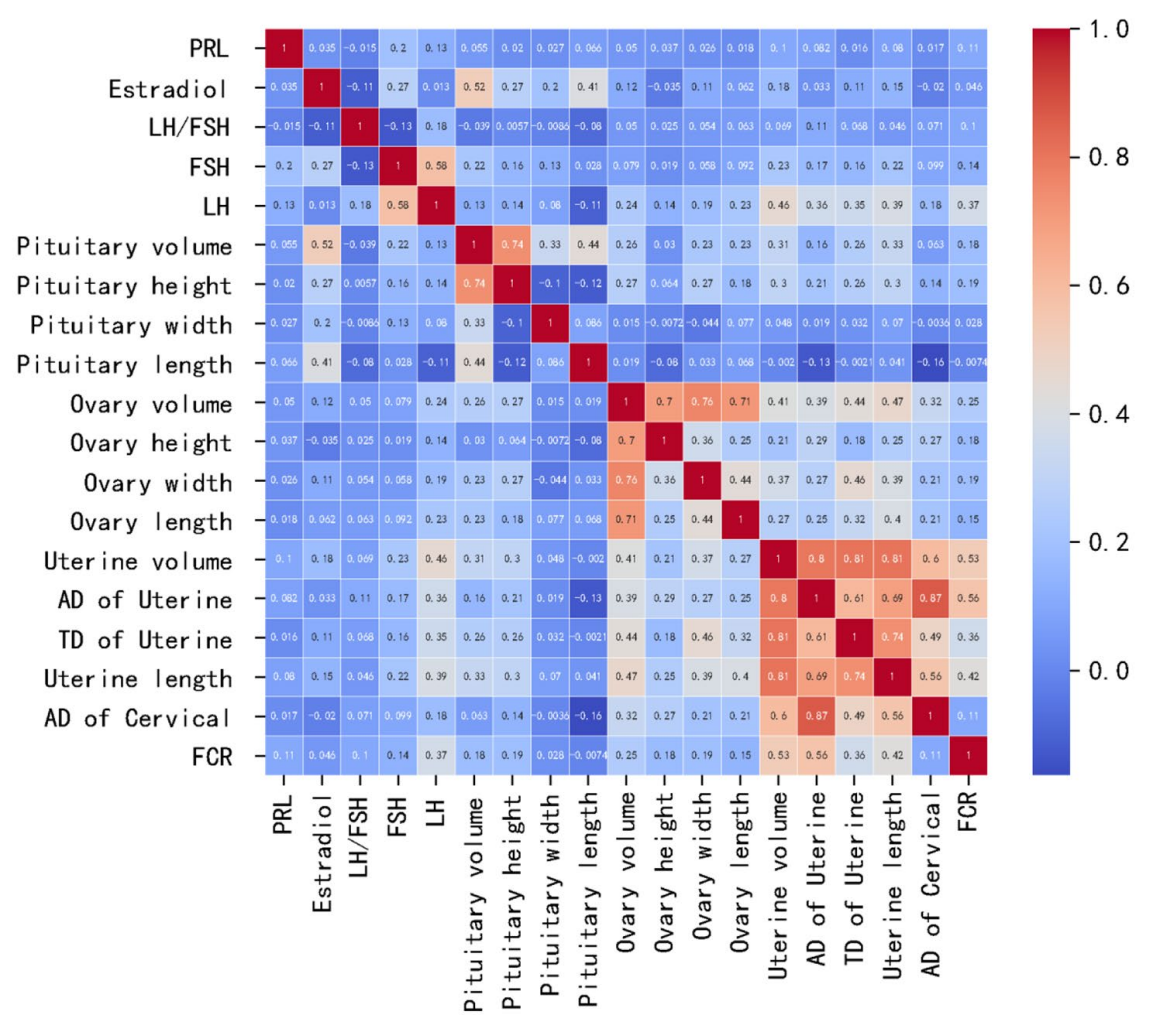
Correlation of the pelvic ultrasound and pituitary MRI parameters with pubertal hormone levels in ICPP group. ∗Values in black font represent statistically significant correlations. Ovarian parameters are mean values for bilateral ovaries Abbreviations: PRL: prolactin; LH, luteinizing hormone; FSH, follicle-stimulating hormone; TD, Transverse diameter; AD, Anteroposterior diameter; FCR, Ratio of fundus to cervix

The correlation coefficient was calculated to evaluate the correlation between serum hormone levels and pituitary, uterine or ovary maturation (Fig. 5). Basal LH levels were positively correlated with pituitary volume, pituitary height, uterine length, transverse diameter of uterine, anteroposterior diameter of uterine, uterine volume, cervical anteroposterior diameter, ratio of fundus to cervix, ovarian length, ovarian width, ovarian height, and ovarian volume. Basal FSH levels were positively correlated with uterine length, transverse diameter of uterine, anteroposterior diameter of uterine, uterine volume, pituitary height, pituitary width, and pituitary volume. The estradiol levels were positively correlated with pituitary volume, pituitary height, pituitary width, pituitary length, uterine volume, and uterine length (all *P* < 0.05).

## Discussion

The GnRHa stimulation test is the gold standard for diagnosing ICPP. However, it has the disadvantages of being time-consuming, and causing discomfort to the patient. This study developed a machine-learning based diagnostic model using basal hormone laboratory values, pituitary parameters and pelvic ultrasound variables for predicting response to GnRHa stimulation testing. Of the four machine learning models, the XGBoost model had best diagnostic efficacy in the internal validated dataset, with an AUC of 0.81, a sensitivity of 81%, and a specificity range of 72%.

Attempts have been made to find other alternatives to GnRHa stimulation tests that are more acceptable and widely available, including serum basal gonadotropin levels and imaging studies [14, 18, 19]. Some investigators have reported basal LH > 0.1–0.83 IU/L as a reliable diagnostic cut-off for CPP with 64–93% diagnostic sensitivity from ICMA sample [18, 20–22]. Previous studies have suggested that independent basal FSH or basal LH/FSH ratios have weak discriminatory power, largely due to the significant overlap in FSH levels between girls with and without CPP [18, 20, 22–24].

Several studies have employed scoring systems or machine learning models to diagnose ICPP. ah et al. incorporated clinical information and a simplified GnRHa test (hormone levels at 30 min post-stimulation only) to construct a machine learning model and reported excellent diagnostic efficacy, with an F1 score of 0.976 and an AUC of 0.972. However, this still did not entirely obviate the need for a GnRHa test to be performed [25]. A practical scoring system based on breast Tanner stage, basal LH and basal FSH was developed with a sensitivity of 76% and a specificity of 72% [26]. A comparable scoring system demonstrated higher efficacy in another study [27], however neither included commonly used imaging parameters in the model.

Brain MRI is usually used to detect CPP-related anatomical abnormalities rather than as a diagnostic indicator [28, 29]. A recent study explored the diagnostic efficacy of pituitary dimension for CPP girls aged 2–8 years, and found a low sensitivity(54.10%) and specificity(72.20%) of pituitary volume at the cut-off value of 196.01mm^3^ [19]. A small sample for pituitary MRI radiomics study with a total of 30 individuals reported moderate diagnostic efficacy with an AUC of 0.76 [30]. However, a poor performance of pituitary imaging histology was reported in another large-sample study (AUC < 0.70) and MRI-related parameters were still included in their model ultimately [31]. Our results demonstrated a large overlap of pituitary MRI parameters between the two groups, which is consistent with previous findings. Considering the current controversy about the necessity of MRI in pubertal children, our diagnostic model ultimately did not incorporate MRI-related parameters.

Pelvic ultrasonography is a rapid, non-invasive, and low-cost examination to assess uterine and ovarian development. uterine and ovarian measurements could help to distinguish girls with CPP and isolated premature thelarche [14, 32, 33]. A recent meta-analysis noted that uterine length and volume are important markers for differentiating CPP from premature thelarche, e.g., a uterine length of 3.2 cm had a diagnostic AUC of 0.82, with a sensitivity and specificity of 81.8% and 82.0%, respectively [11]. FCR is regarded as a crucial indicator of puberty. However, past investigations have yielded inconsistent findings [13, 34, 35]. Recently, a comprehensive meta-analysis identified a notable disparity in FCR between the CPP and non-CPP groups, signifying the potential of this parameter in effectively discriminating CPP [11]. Our result further underscores its pivotal diagnostic utility. Therefore, we incorporated ultrasound parameters with clinical information into the machine learning model.

Our model shows good efficacy in the validation set with an AUC of 0.81 and an F1 score of 0.80. The model incorporates several predictors including basal LH, FSH, estradiol levels and LH/FSH ratio, averaged ovarian volume, and several uterine parameters. According to our machine learning model, basal LH, FSH, estradiol levels and LH/FSH ratio are among the top ten predictors of feature importance in the XGBoost classifier. These pubertal hormones, as important products during the activation of the hypothalamic-pituitary-gonadal axis, are the endocrine basis of CPP. Among the ovarian parameters, only mean ovarian volume was a significant predictor. This may be due to the irregularity of ovarian morphology resulting in high variability of single radial values [11].

The positive correlations between pituitary, uterine and ovarian volumes, and pubertal hormones were found, while part of the correlations was relatively weak. For pituitary development, the influence of hormones other than HPG-axis hormones on the pituitary volume cannot be ignored [36]. For instance, enlarged pituitary glands has been reported to be associated with relatively high levels of dehydroepiandrosterone (DHEA) and its sulphate (DHEA-S) in children [37]. For the development of uterine and ovarian, besides pubertal hormones, other factors such as genetics and the environment also play an important role [38].

Our study had some limitations. First, as a cross-sectional study, the growth velocity was not fully documented, and future studies need to focus on the impact of this metric on pubertal children. Second, our ultrasound parameters did not include indicators such as cervical length, quantity of large follicles, and maximum follicular diameter. This is because some of the ultrasound reports had incomplete descriptions of these parameters. In addition, recent meta-analyses have pointed out that these indicators are not yet reliable predictors [9, 11]. Finally, our current study only focused on female patients with ICPP. With the increasing incidence of male precocious puberty, future research should also focus on simplifying the diagnosis of male precocious puberty.

## Conclusions

In this study, we developed a machine learning model incorporating ultrasound parameters, underlying hormones and clinical information applied to CPP diagnosis. The model had good performance in predicting the response to stimulation testing prior to GnRHa injection in girls who are suspected CPP.

## Data Availability

The analyzed data sets generated during the study are available from the corresponding author on reasonable request.

## Abbreviations

GnRHa: Gonadotropin-releasing hormone analogue
HPG: Hypothalamic-pituitary-gonadal axis
CPP: Central precocious puberty
LH: Luteinizing hormone
FSH: Follicle-stimulating hormone
MRI: Magnetic resonance imaging
FCR: Ratio of fundus to cervix
ROC: Receiver operating characteristic curve
AUC: Area under the curve. LightGBM:light gradient boosting, XGBoost:eXtreme gradient boosting
SHAP: SHapley Additive exPlanations
PPV: Positive predictive value
NPV: Negative predictive value
DCA: Decision curve analysis
